# Bullying/Victimization Experiences, Post-Traumatic Stress Disorder, Mental Resilience and Self-Compassion in Adolescent Students

**DOI:** 10.1007/s40653-026-00921-3

**Published:** 2026-06-04

**Authors:** Vasiliki Makrydaki, Eleni Andreou

**Affiliations:** Department of Primary Education, University of Thessaly, Volos, Greece

**Keywords:** Bullying/victimization, Adolescents, PTSD, Mental resilience, Self-compassion

## Abstract

The present study was designed to investigate the association between post-traumatic stress, mental resilience and self-compassion in adolescents involved in bullying incidents at school. It also examined whether there are differences between: a) boys and girls, and b) victims, bullies, bully/victims, observers and students non-involved in bullying, in terms of levels of post-traumatic stress, resilience and self-compassion. A total of 658 students, 277 boys (42%) and 381 girls (58%), attending the first grade of 17 different high schools in Thessaly, Greece, participated in the study by completing a series of self-report questionnaires. The results indicated higher PTSD symptom scores for girls compared to boys, whereas boys reported higher total self-compassion (primarily reflecting lower self-judgment). Moreover, victims and bully/victims showed higher levels of PTSD compared to bullies, observers, and students non-involved in bullying. Observers showed higher levels of mental resilience compared to victims and bully/victims. Observer and non-involved students showed higher levels of self-compassion compared to victims and bully/victims. Finally, mental resilience was positively correlated to self-compassion, and total PTSD symptoms were negatively correlated to mental resilience and self-compassion. These findings may significantly contribute toward designing interventions for strengthening mental resilience and cultivating self-compassion to address bullying.

## Introduction

According to the well-known definition, school bullying refers to the implicit and repeated aggression by one student (bully) against another student (victim), between whom there is an inequality of power (Juvonen & Graham, 2014; Olweus, 1993). Forms of school bullying are verbal (e.g., name-calling), physical (e.g., kicking and hitting), relational (e.g., rumor spreading and social exclusion), and virtual (i.e. cyberbullying through messages and/or social media posts; Olweus et al., 2019; Rettew & Pawlowski, 2016).

A more current, revised definition of school bullying and cyberbullying refers to the damaging social process that is characterized by an imbalance of power driven by social norms (O’Higgins Norman et al., 2024). This implies the absence of effective response and care from peers or adults to victims, and the involvement of perpetrators in bullying because of group dynamics rather than their intention to harm the victim. Additionally, bullying is repeated. Repetition is not only linked to the number of times that incidents of bullying occur, but also, it also relates to the effect on the victim who may fear that a one-off event might be repeated or re-shared online. Finally, bullying manifests as an unwanted interpersonal behavior among students that causes physical, social, and emotional harm to the victim(s) and the wider school community (O’Higgins Norman et al., 2024).

### Posttraumatic Stress Disorder in Bullying

Indicatively, some negative consequences identified for victims include high levels of anxiety (Garnefski & Kraaij, 2014; Valera-Pozo et al., 2021), social phobia, and post-traumatic stress disorder (PTSD; Andreou et al., 2021; Kurniawan et al., 2022; Makrydaki et al., 2024; Moore et al., 2017). PTSD concerns a specific pattern of symptoms because of a traumatic event, such as re-experiencing trauma memories, avoidance of cues that remind the individual of the trauma, negative emotions and thoughts, and hyperarousal (APA, 2013). PTSD symptoms later in bullying victims’ lives suggest that bullying is a form of interpersonal trauma (Evans et al., 2017). Idsoe et al. (2021) suggested that Developmental Trauma Disorder and Complex PTSD better capture the complexity of the symptoms associated with bullying, than PTSD.

The level of PTSD symptoms among the bullied children is high, especially within the groups of girls, and bully/victims (Idsoe et al., 2012). The research on bullying makes a distinction between victims, bullies, and bully/victims, which refers to youth who are involved in both perpetration and victimization. In particular, Kennedy (2021) highlights that there is significant diversity within the group of youth who experience both perpetration and victimization in relation to trauma symptoms, i.e. youth, aged 5 to 17 years, who are involved equally in high rates of perpetration and victimization experienced more trauma symptoms than the victimization predominant bully/victims, the aggression predominant bully/victims, and the bully/victims who only occasionally involved in perpetration and victimization. However, other research does not support gender differences in PTSD symptoms (Andreou et al., 2021) or differences between victims and bully/victims (Andreou et al., 2021; Sterzing et al., 2020).

### Self-compassion in Victims and Bully/Victims

The results from a longitudinal design study showed that stressful bullying victimization weakens self-compassion, leading to trauma-related shame; thus, influencing college students to exhibit more cyber-aggression and less prosocial behavior (Xu et al., 2024). However, this study did not examine the differences between bullying victimization roles (e.g. victims, bullies, bully/victims). Adolescent victims with low self-compassion are more likely to experience feelings of anxiety (Chu et al., 2018). Even though a great deal of the literature on school bullying has focused on negative consequences for victims and bully/victims, such as PTSD symptoms, a growing body of literature has paid attention to victims’ self-compassion.

Self-compassion entails being kind and understanding toward oneself in instances of pain or failure rather than being harshly self-critical, perceiving one’s experiences as part of the larger human experience rather than seeing them as separating and isolating, and holding painful thoughts and feelings in balanced awareness, rather than over-identifying with them (Neff, 2003). Self-criticism is a strong predictor of the role of victim of bullying, i.e. the adolescent with a high level of self-criticism explains to themselves the experience of bullying as their own fault. This often leads to the self being seen as defective, inappropriate, and undesirable; therefore, they are less likely to be able to overcome the position of the victim (Zăbavă, 2020). One possible explanation of the negative association between bullying and self-compassion is that growing up in a hostile peer environment may hinder the development of self-compassion and erode the capacity of treating oneself kindly (Múzquiz et al., 2023).

On the other hand, adolescents with higher levels of self-compassion show more kindness toward themselves may be able to understand their victimization experiences, rather than harshly criticizing or blaming themselves. Therefore, self-compassion is an adaptive coping mode, which may allow them to cope more successfully with painful thoughts and feelings resulting from victimization (e.g., positive cognitive restructuring; Chu et al., 2018). Similarly, university students who were former victims of bullying with high self-compassion feel less shame about the experience of bullying victimization in adulthood (Beduna & Perrone-McGovern, 2019). In addition, a longitudinal study on victimized adolescents in China found that self-compassion mediated the relationship between victimization and depression in victims, i.e., victims with greater self-compassion treat themselves better when they are bullied (Yan et al., 2022). However, this research has limited external validity, as it focused on victimized adolescents from China, who lived in rural areas with their grandparents.

### Mental Resilience in Victims and Bully/Victims

Research has shown that children and adolescents with a higher level of resilience experience fewer symptoms of anxiety under bullying victimization, i.e. resilience plays a prominent mediating role in the association between bullying victimization and anxiety (Fang et al., 2022). Resilience is a dynamic process of effectively negotiating, adapting to, or managing significant sources of stress or trauma (Rutter, 2006). Concerning resilience, boys reported higher levels than girls (Andreou et al., 2020; Saaduddin et al., 2023), and bully/victims were found to be less resilient compared to victims and bullies (Bunnett, 2021).

Švecová et al. (2023) supported that the protective effect of resilience was moderated by bullying in several types of psychopathologies; specifically, the respondents who were bullied had higher scores in somatization, obsessive-compulsive disorder, interpersonal sensitivity, depression and psychoticism. On the other hand, a longitudinal study found that adolescents who have experienced bullying, both as victims and bully/victims, usually have a higher risk of depression and suicidal thoughts and that resilience can offer protection against depression (Lin et al., 2022).

Moreover, victims’ perceived social/family support, interpersonal assistance (Fang et al., 2022; Makrydaki et al., 2024), personal assets, emotional regulation (Fang et al., 2022), social competence, structured style, self-reliance, and optimism about the future, seemed to act as a counterbalance to the traumatic victimization they experienced and as an essential protective factor against PTSD symptoms (Makrydaki et al., 2024). Additionally, self-esteem affects the resilience of students who are victims of bullying, i.e. students with high self-esteem are more able to face challenges and failures and confident in overcoming difficulties (Saaduddin et al., 2023; Sapouna & Wolke, 2013).

Moreover, self-efficacy, social support, and resilience were found to partially mediate the effect of previous bullying experience on current well-being and mental illness in university students from China and Germany (Lin et al., 2020). A large number of studies have focused on former victims of school bullying concerning their mental resilience (e.g. Lin et al., 2020; Makrydaki et al., 2024; Švecová et al., 2023) and self-compassion (e.g. Beduna & Perrone-McGovern, 2019; Xu et al., 2024). Although a clear moderating effect of resilience on the relationship between students’ victimization and well-being cannot be claimed (Andreou et al., 2020), resilience has been linked to other positive outcomes after the bullying experience. To the authors’ knowledge, only one study has investigated the association between bullying victimization, mental resilience, and self-compassion in students, which is limited to 130 junior high school participants of two schools in Bangkok metropolitan area, with females constituting the majority of the sample (Bowornkittikun et al., 2021).

### The Present Study

The present quantitative study was designed to address the above gaps in the literature by exploring the association between post-traumatic stress, mental resilience, and self-compassion in adolescents involved in bullying incidents at school. It also examined the main and interaction effects of gender (boys vs. girls) and bullying role (victims, bullies, bully/victims, observers, and non-involved students) on PTSD, resilience, and self-compassion. A negative association between PTSD symptoms with mental resilience and self-compassion was expected. Moreover, it was hypothesized that girls would experience higher levels of PTSD symptoms than boys (Idsoe et al., 2012), while boys would experience higher levels of mental resilience and self-compassion than girls (e.g. Saaduddin et al., 2023). Differences in mental resilience by gender were examined exploratorily. Victims and bully/victims were also expected to experience more PTSD symptoms than other groups of students (Andreou et al., 2021; Sterzing et al., 2020), while non-involved students were expected to exhibit higher levels of mental resilience and self-compassion than the other groups of students (Bunnett, 2021).

## Method

### Participants

A total of 658 students, 277 boys (42%) and 381 girls (58%), attending the first grade of 17 different high schools in Thessaly, Greece, participated in the study by completing a series of self-report questionnaires. Of all participants, 30% (*n* = 196) were identified as victims by their responses regarding frequency of reported physical and verbal bullying, harassment, and theft of personal belongings at school, 9% (*n* = 56) as bullies, 19% (*n* = 127) as bully/victims, 27% (*n* = 180) as observers, and 15% (*n* = 99) as non-involved in bullying incidents.

### Measures

#### School Bullying

The questionnaire “Exposure to Aggression and Violence incidents at School, in the Neighborhood and at Home Checklist” developed by Makri-Botsari (2010) was used to explore school bullying experiences. The scale consists of 75 multiple-choice questions, which explore experiences of aggressive/violent behaviors that someone has either received as a victim, or manifested as bully against others, or witnessed as an observer at school, home, or in the neighborhood. Responses were related to the frequency [4-point scale; *never* (0), *only once* (1), *sometimes* (2), *often* (3)] with which students were involved in physical (e.g., “How often have you been badly beaten up at your school?”), verbal attacks (e.g., “At your school, how often have you been verbally or emotionally abused or insulted and made to feel afraid or uncomfortable?”), and in actions such as theft of personal belongings (e.g., “Have you been robbed at school?”). For the purposes of this study, we were only interested in frequency of participants’ involvement in bullying (aggression/violence) incidents at school as victims, bullies, bully/victims, observers, and non-involved students.

Based on participants’ self-reports, students were classified into five mutually exclusive bullying-related roles, following the procedures used by Dodge et al. (1997) and Makri-Botsari (2010). Specifically, a respondent was considered a victim if they reported being bullied in one or more of the above ways “*sometimes*” or “*often*” and did not report bullying others at the same frequency. A respondent was considered a bully if they reported having bullied others in one or more of the above ways “*sometimes*” or “*often*” and did not report being bullied at the same frequency. A respondent was considered a bully/victim if they reported both being bullied and having bullied others in one or more of the above ways “*sometimes*” or “*often*”. Students were classified as observers if they reported having observed physical and/or verbal bullying incidents “*sometimes*” or “*often*” in the relevant eight items of the questionnaire and did not meet criteria for victim, bully, or bully/victim. Finally, students were considered non-involved if they did not report having been bullied, having bullied others, or having observed bullying incidents at the above frequency thresholds.

For this sample, the internal consistency of the role-defining subscales was strong: (a) victimization (*Cronbach’s α* = 0.78), (b) bullying perpetration (*Cronbach’s α* = 0.85), and (c) observing (*Cronbach’s α* = 0.83). The bully/victim and non-involved categories were derived using rule-based cut-offs applied to these subscale scores and therefore do not represent separate subscales.

#### Post-Traumatic Stress Disorder Symptoms

Symptoms of PTSD were assessed using the “Children’s Post-Traumatic Stress Disorder-Reaction Index” (CPTSD-RI) (Pynoos et al., 1987), which was adapted in Greek by Kolaitis et al. (2011). The questionnaire contains 20 questions that compose three factors: “intrusiveness/numbing/avoidance”, “fear/anxiety symptoms”, and “disturbances in sleep and concentration”. Items are rated on a 4-point scale ranging from *none of the time* (0) to *most of the time* (4). Higher scores indicate more severe PTSD symptoms. In the present study, Principal Component Analysis (PCA) suggested three components:1) “intrusive thoughts, numbing and hyperarousal” (12 items, e.g., “Repeated, disturbing, and unwanted memories of the bullying experience?”), 2) “negative rumination”, i.e. negative changes in cognitive functions/disposition resulting from “intentional” thinking, not from intrusive thinking (5 items, e.g., “Feeling very scared or upset when you thinking about bullying experience?”) and 3) “changes in daily life”, such as disturbances in sleep and concentration (3 items e.g., “Loss of interest in activities that you used to enjoy after the school bullying incident(s)?”). The overall *Cronbach’s alpha* was strong (0.89). The internal consistency of the sub-scales was: *Cronbach’s alpha* = 0.91 for “intrusive thoughts, numbing and hyperarousal”, 0.71 for “negative rumination”, and 0.52 for “changes in daily life”. Given its low internal consistency (*α* = 0.52), the “changes in daily life” subscale was excluded from subsequent subscale-level analyses and was not interpreted further. The CPTSD-RI total score was retained as the primary PTSD indicator in the main analyses, whereas the two more reliable PTSD subscales were examined only in supplementary analyses.

#### Mental Resilience

An adapted version of the Resilience Scale (Wagnild & Young, 1993), modified by Neill and Dias (2001), and translated into Greek by Leontopoulou (2010), was used to measure mental resilience. The scale consists of 15 items that are positively worded, and responses ranged from *agree* (1) to *disagree* (7). In the present study, we conducted a Principal Component Analysis (PCA) to examine the component structure in our sample; the analysis suggested two components : (1) “personal strength and structured style” (9 items, e.g. “I have self-discipline”) and (2) “self-efficacy and ability to achieve” (6 items, e.g. “Manage one way or other”). In the present study, the total resilience score was treated as the primary indicator of overall resilience, whereas PCA-derived component scores were examined only in supplementary analyses. In the present study, the overall *Cronbach’s alpha* was strong 0.92. The internal consistency of the sub-scales was also strong (*Cronbach’s alpha* = 0.89 for “personal strength and structured style”, and 0.83 for “self-efficacy and ability to achieve”).

#### Self-Compassion

Participants were given the Greek version of the 26-item Self-Compassion Scale (SCS) (Neff, 2003), which was translated by Mantzios et al. (2015). The SCS includes the 5-item “self-kindness” sub-scale, the 5-item “self-judgment” sub-scale, the 4-item “common humanity” sub-scale, the 4-item “isolation” sub-scale, the 4-item “mindfulness” sub-scale, and the 4-item “over identification” sub-scale. Responses are given on a 5-point scale, *almost never* (1) to *almost always* (5). Negatively worded items were reverse-coded prior to scoring so that higher values consistently indicated greater self-compassion. An overall self-compassion total score was computed as the sum of all 26 items (range: 26–130), with higher scores indicating greater levels of self-compassion. Therefore, the total score integrates all six original SCS subscales, with the negatively worded subscales/items reverse-coded so that higher total scores reflect higher levels of self-kindness, common humanity, and mindfulness, and lower levels of self-judgment, isolation, and over-identification.

In the present study, this total score was treated as the primary indicator of overall self-compassion. To further clarify the total-score findings across gender and bullying-role groups (victims, bullies, bully/victims, observers, and non-involved students), we also examined PCA-derived component scores in this sample. These component scores were used as supplementary indicators and should not be interpreted as equivalent to the original six SCS subscales. Principal Component Analysis (PCA) suggested four components: (1) “low self-judgment” (13 items, e.g. “I’m disapproving and judgmental about my own flaws and inadequacies”), (2) “self-kindness” (6 items e.g. “I try to be loving towards myself when I’m feeling emotional pain”), (3) “common humanity” (4 items, e.g. “When I’m down and out, I remind myself that there are lots of other people in the world feeling like I am”) and (4) “mindfulness” (3 items, e.g. “When something upsets me, I try to keep my emotions in balance”). To facilitate interpretation, the reverse-scored negative component is reported as “low self-judgment”; thus, higher scores reflect lower self-criticism, consistent with the scoring direction of the total self-compassion score. In the present study, the overall *Cronbach’s alpha* was strong (0.79). The internal consistency of most PCA-derived components was satisfactory to strong: 0.91 for “low self*-*judgment (reverse-scored)”, 0.82 for “self-kindness” and 0.72 for “common humanity”, except for “mindfulness” which had a *Cronbach’s alpha* of 0.65, potentially because it only has 3 items.

### Research Design

An invitation letter was sent to school principals requesting participation of students in the study. The letter fully explained to principals and teachers the purpose of the study. The educational staff of each school that was interested and agreed, communicated with the researchers and arranged an in-person meeting between teachers, students, and researchers in order to inform the students about the research purpose. The parents (and their children) who were interested read and signed a consent form, which briefly explained the rationale of the research and informed them about their children’s anonymity. Data were collected online in the computer lab of each school. Ethical approval was obtained by the responsible Ethics Committee.

To examine score organization in the present sample and to support score derivation for supplementary analyses, we conducted Principal Component Analyses (PCA) for the CPTSD-RI, the Resilience Scale, and the Self-Compassion Scale. Although these measures have established structures in prior research and Greek translations/adaptations were available, they were originally developed in different linguistic/cultural contexts and, to our knowledge, their component structure had not been established in school-based adolescent sample PCA was therefore used as a pragmatic data-reduction procedure to obtain sample-specific component scores, rather than to replace the original theoretical structure or estimate a latent variable model. The original total scores were retained as the primary variables in the main analyses, whereas PCA-derived component scores were examined only in supplementary analyses to clarify specific group differences and patterns of association. Across PCAs, Varimax rotation with Kaiser normalization was applied; components were retained based on eigenvalues > 1 and scree-plot inspection, and salient loadings were interpreted at |loading| ≥ 0.40.

Statistical analyses were performed using IBM SPSS Statistics 26. To examine group differences by gender and bullying role, a 2 (Gender: boys, girls) × 5 (Bullying role: victims, bullies, bully/victims, observers, non-involved) factorial multivariate analysis of variance (MANOVA) was conducted on the total scores of PTSD, mental resilience, and self-compassion. To further clarify these findings, supplementary univariate analyses were conducted for the two more reliable PTSD subscales, whereas additional factorial MANOVAs were conducted separately for the resilience subscales and the PCA-derived self-compassion components. Assumptions were evaluated prior to interpretation; because Box’s M tests were significant, multivariate effects were interpreted using Pillai’s Trace. Where multivariate effects were significant, follow-up univariate ANOVAs were conducted. Pairwise comparisons and simple-effects tests were based on estimated marginal means with Bonferroni adjustment. Pearson’s correlations were computed to examine associations among PTSD symptoms, resilience, and self-compassion. Descriptive statistics reported in tables represent observed (raw) means and standard deviations.

## Results

### Effects of Gender and Bullying Role on PTSD, Resilience, and Self-Compassion (MANOVA)

#### Total Scores

A two-way MANOVA (gender × bullying role) was conducted with the total scores of PTSD, resilience, and self-compassion scales as dependent variables. Observed (raw) means and standard deviations, along with the follow-up univariate tests, are presented in Table 1 (gender) and Table 2 (role). Box’s M test was significant (*p* < .001); therefore, multivariate effects were interpreted using Pillai’s Trace. Levene’s tests suggested heterogeneity of variances for all three dependent variables (*ps* < 0.001); therefore, follow-up univariate results were interpreted cautiously, especially given unequal cell sizes. Bonferroni adjustments were used to control family-wise error in multiple comparisons. The MANOVA indicated statistically significant multivariate effects of gender (Pillai’s Trace = 0.03, *F*(3, 646) = 6.23, *p* < .001, ηp² = 0.03), role (Pillai’s Trace = 0.18, *F*(12, 1944) = 10.20, *p* < .001, ηp² = 0.06), and gender × role (Pillai’s Trace = 0.04, *F*(12, 1944) = 2.19, *p* = .010, ηp² = 0.01).

**Table 1:** Gender Differences in PTSD Symptoms, Mental Resilience, and Self-Compassion

Measure	Total (*N* = 658)		Boys (*N* = 277)		Girls (*N* = 381)		F(1,648)	*p*	ηp^2^
	M	SD	M	SD	M	SD			
Post-traumatic stress	38.45	12.41	36.35	11.50	39.97	12.84	9.54 *	0.002	0.02
Intrusive thoughts, numbing, hyperarousal	19.40	8.61	18.03	7.50	20.39	9.21	9.36 *	0.002	0.01
Negative rumination	10.93	3.69	9.92	3.42	11.66	3.71	17.32 *	< 0.001	0.03
Mental resilience	80.53	16.74	79.92	19.65	80.97	14.27	1.67	0.197	0.00
Personal strength and structured style	46.59	11.22	47.14	12.36	46.20	10.32	0.01	0.937	0.00
Self-efficacy, ability to achieve	33.94	6.68	32.78	8.11	34.78	5.26	11.44 *	< 0.001	0.02
Self-compassion	77.35	13.84	79.36	10.15	75.89	15.85	5.90 *	0.015	0.01
Low self-judgment	41.08	12.19	44.18	11.23	38.83	12.38	19.09 *	< 0.001	0.03
Self-kindness	16.86	5.64	16.16	5.73	17.37	5.53	3.73	0.054	0.01
Common humanity	10.65	3.84	10.34	3.98	10.87	3.73	1.84	0.176	0.00
Mindfulness	8.76	3.04	8.68	3.17	8.82	2.95	0.57	0.451	0.00

*p < .05. Note. Values are observed (raw) means and standard deviations. Inferential tests were conducted using a 2 (Gender) × 5 (Role) MANOVA; pairwise comparisons and simple-effects tests were based on estimated marginal means with Bonferroni adjustment.

Univariate follow-ups showed that gender differences emerged for PTSD (*F*(1, 648) = 9.54, *p* = .002, ηp² = 0.02) and self-compassion (*F*(1, 648) = 5.90, *p* = .015, ηp² = 0.01), but not resilience (*p* = .197). Girls reported higher PTSD than boys (Bonferroni-adjusted *p* = .002), whereas boys reported higher self-compassion than girls (Bonferroni-adjusted *p* = .015).

**Table 2:** Differences in PTSD Symptoms, Mental Resilience, and Self-compassion Based on Bully or Victim Status

Measure			Total (*N* = 658)				Victims (*N* = 196)			Bullies (*N* = 56)						Bully/Victims (*N* = 127)						Observers (*N* = 180)			Students not *involved* (*N* = 99)				Role F(4,648)	*p*	ηp^2^
			M			SD	M		SD	M				SD		M				SD		M	SD			M		SD			
1	38.45				12.41			42.19^cde^	12.34	35.46^c^			10.73		44.48^abde^				12.65		34.66^ac^			9.12			31.87^ac^	12.55	25.12 *	< 0.001	0.13
2	19.40				8.61			21.39^cde^	9.27	17.89^c^			6.95		23.62^abde^				9.64		16.48^ac^			5.70			16.17^ac^	7.83	21.01 *	< 0.001	0.12
3	10.93				3.69			12.16^bde^	3.72	9.55^ac^			3.17		12.07^bde^				3.80		10.18^ac^			2.93			9.15^ac^	3.73	17.04*	< 0.001	0.10
4	80.53				16.74			79.27^d^	15.48	81.04			20.50				76.23^d^	17.37			85.22^ac^		12.29			79.73		20.82	6.37 *	< 0.001	0.04
5	46.59				11.22			45.06^d^	11.03	48.48^c^			11.90				43.66^bd^	11.63			49.53^ac^		8.95			46.99		13.01	7.22 *	< 0.001	0.04
6	33.94				6.68			34.21	5.69	32.55			9.17				32.57^d^	7.21			35.69^ce^			4.54			32.74^d^	8.52	4.76 *	< 0.001	0.03
7		77.35		13.84			74.51^de^		15.95		79.27^c^	10.61				73.04^bde^			12.11		80.57^ac^			13.17			81.55^ac^	11.41	11.32 *	< 0.001	0.07
8	41.08				12.19			37.61^de^	11.95	43.30			10.02				38.99^de^		10.94		42.58^ac^		11.53			46.63^ac^		13.83	10.19 *	< 0.001	0.06
9	16.86				5.64			17.01	5.41	16.43			5.87				15.74^d^		5.36		17.88^c^		5.19			16.39		6.75	2.56 *	0.037	0.02
10	10.65				3.84			10.90	3.82	10.70			4.00				10.06		3.76		11.01		3.61			10.23		4.22	1.57	0.181	0.01
11	8.76				3.04			8.99	2.92	8.84			3.24				8.24		3.08		9.09		2.84			8.29		3.38	2.77*	0.027	0.02

*p < .05. 1: Post-traumatic stress, 2: Intrusive thoughts, numbing, hyperarousal, 3: Negative rumination, 4: Mental resilience, 5: Personal strength and structured style, 6: Self-efficacy, ability to achieve, 7: Self-compassion, 8: Low Self-Judgment, 9: Self-kindness, 10: Common humanity, 11: Mindfulness. a: significant difference (p<.05) from the victims’ group, b: significant difference (p<.05) from the bullies’ group, c: significant difference (p<.05) from the bully/victims’ group, d: significant difference (p<.05) from the observers’ group (2-tailed tests) and e: significant difference (p<.05) from the students’ not involved group.

Note Values are observed (raw) means and standard deviations. Inferential tests were conducted using a 2 (Gender) × 5 (Role) MANOVA; pairwise comparisons and simple-effects tests were based on estimated marginal means with Bonferroni adjustment.

Role differences were statistically significant for all dependent variables (PTSD: *F*(4, 648) = 25.12, *p* < .001, ηp² = 0.13; resilience: *F*(4, 648) = 6.37, *p* < .001, ηp² = 0.04; self-compassion: *F*(4, 648) = 11.32, *p* < .001, ηp² = 0.07). Bonferroni-adjusted comparisons indicated that bully/victims reported higher PTSD than victims (*p* = .009), bullies (*p* < .001), observers (*p* < .001), and students not involved (*p* < .001), while victims reported higher PTSD than observers and students not involved (*ps* < 0.001). For resilience, observers scored higher than victims (*p* = .018) and bully/victims (*p* < .001). Bonferroni-adjusted comparisons for self-compassion indicated that victims scored lower than observers (*p* = .004) and students not involved (*p* = .004), and bully/victims scored lower than bullies (*p* = .014), observers (*p* < .001), and students not involved (*p* < .001).

The gender × role interaction was statistically significant only for self-compassion, *F*(4, 648) = 3.86, *p* = .004, ηp² = 0.02. Simple effects analyses with Bonferroni adjustment indicated that boys reported higher self-compassion than girls among victims (boys: *M* = 77.64; girls: *M* = 73.36; mean difference = 4.29, *p* = .044) and especially among bully/victims (boys: *M* = 77.76; girls: *M* = 66.23; mean difference = 11.53, *p* < .001). Gender differences were not statistically significant among bullies, observers, or not-involved students (*ps* ≥ 0.526). This interaction should be interpreted cautiously due to unequal cell sizes (notably girls–bullies *n* = 12).

### Supplementary Analyses of the two Retained PTSD Subscales

Given the exclusion of the “changes in daily life” subscale due to its low internal consistency, supplementary univariate analyses were conducted for the two more reliable PTSD subscales, “Intrusive thoughts, numbing, hyperarousal” and “Negative rumination.” Girls scored higher than boys on both retained PTSD subscales: “Intrusive thoughts, numbing, hyperarousal” (*F*(1, 648) = 9.36, *p* = .002, ηp² = 0.01) and “Negative rumination” (*F*(1, 648) = 17.32, *p* < .001, ηp² = 0.03).

Role differences were also significant for both retained PTSD subscales. For “Intrusive thoughts, numbing, hyperarousal”, *F*(4, 648) = 21.01, *p* < .001, ηp² = 0.12, bully/victims scored higher than victims, bullies, observers, and students not involved (all Bonferroni-adjusted *ps* < 0.001), whereas victims scored higher than observers (*p* < .001) and students not involved (*p* = .002). For “Negative rumination”, *F*(4, 648) = 17.04, *p* < .001, ηp² = 0.10, victims scored higher than bullies (*p* = .009), observers (*p* < .001), and students not involved (*p* < .001), and bully/victims scored higher than bullies, observers, and students not involved (all Bonferroni-adjusted *ps* < 0.001), while bully/victims did not differ from victims (*p* = .822).

The gender × role interaction was significant only for “Intrusive thoughts, numbing, hyperarousal”, *F*(4, 648) = 2.96, *p* = .019, ηp² = 0.02. Simple effects analyses with Bonferroni adjustment indicated that girls reported higher “Intrusive thoughts, numbing, hyperarousal” than boys among victims (boys: *M* = 17.21; girls: *M* = 22.94; mean difference = 5.73, *p* < .001) and among bully/victims (boys: *M* = 21.80; girls: *M* = 26.25; mean difference = 4.45, *p* = .002). Gender differences were not significant among bullies, observers, or not-involved students (*ps* ≥ 0.283). This interaction should be interpreted cautiously due to unequal cell sizes (notably girls–bullies *n* = 12).

### Mental Resilience Subscales

A two-way MANOVA (gender × bullying role) was conducted with “Personal strength and structured style” and “Self-efficacy, ability to achieve” as dependent variables. Box’s M test was significant (*p* < .001); therefore, multivariate effects were interpreted using Pillai’s Trace. Levene’s tests suggested heterogeneity of variances for both dependent variables (*ps* < 0.001); therefore, follow-up univariate results were interpreted cautiously, especially given unequal cell sizes. Bonferroni adjustments were used to control family-wise error in multiple comparisons. The MANOVA indicated statistically significant multivariate effects of bullying role (Pillai’s Trace = 0.07, *F*(8, 1296) = 5.87, *p* < .001, ηp² = 0.04) and gender (Pillai’s Trace = 0.04, *F*(2, 647) = 13.35, *p* < .001, ηp² = 0.04). The gender × role interaction was not statistically significant (Pillai’s Trace = 0.02, *F*(8, 1296) = 1.37, *p* = .207, ηp² = 0.01).

Regarding gender, differences emerged only for “Self-efficacy, ability to achieve” (*F*(1, 648) = 11.44, *p* < .001, ηp² = 0.02), but not for “Personal strength and structured style” (*p* = .937). Specifically, girls reported higher self-efficacy/ability to achieve than boys (boys: *M* = 32.69; girls: *M* = 34.81; Bonferroni-adjusted *p* < .001).

Univariate follow-ups showed that role differences were statistically significant for both subscales; “Personal strength and structured style”: *F*(4, 648) = 7.22, *p* < .001, ηp² = 0.04; “Self-efficacy, ability to achieve”: *F*(4, 648) = 4.76, *p* < .001, ηp² = 0.03). Bonferroni-adjusted pairwise comparisons (based on estimated marginal means) indicated that for “Personal strength and structured style”, observers scored higher than victims (*p* = .010) and bully/victims (*p* < .001), and bullies scored higher than bully/victims (*p* = .010). For “Self-efficacy, ability to achieve”, observers scored higher than bully/victims (*p* = .002) and students not involved (*p* = .004). The interaction was non-significant; therefore, simple effects were not interpreted as primary findings. Findings should be interpreted cautiously given unequal cell sizes (notably girls–bullies *n* = 12).

### PCA-derived Self-Compassion Components

A two-way MANOVA (gender × bullying role) was conducted with the four PCA-derived self-compassion components– “Low self-judgment” (reverse-scored; higher scores indicate lower self-criticism), “Self-Kindness”, “Common Humanity”, and “Mindfulness” - as dependent variables. Box’s M test was significant (*p* < .001); therefore, multivariate effects were interpreted using Pillai’s Trace. Levene’s tests suggested heterogeneity of variances for all four dependent variables (*ps* < 0.001); therefore, follow-up univariate results were interpreted cautiously, especially given unequal cell sizes. Bonferroni adjustments were used to control family-wise error in multiple comparisons. The MANOVA revealed significant multivariate effects of bullying role (Pillai’s Trace = 0.10, *F*(16, 2592) = 4.03, *p* < .001, ηp² = 0.02) and gender (Pillai’s Trace = 0.03, *F*(4, 645) = 5.68, *p* < .001, ηp² = 0.03), as well as a small role × gender interaction (Pillai’s Trace = 0.04, *F*(16, 2592) = 1.66, *p* = .048, ηp² = 0.01).

Regarding gender, a significant effect emerged only for “Low self-judgment” (reverse-scored), *F*(1, 648) = 19.09, *p* < .001, ηp² = 0.03, with boys reporting lower self-criticism than girls.

Univariate follow-ups indicated significant role differences in “low self-judgment” (reverse-scored), *F*(4, 648) = 10.19, *p* < .001, ηp² = 0.06, “self-kindness”, *F*(4, 648) = 2.56, *p* = .037, ηp² = 0.02, and “mindfulness”, *F*(4, 648) = 2.77, *p* = .027, ηp² = 0.02, whereas “common humanity” was not significant (*p* = .181). Bonferroni-adjusted comparisons (based on estimated marginal means) showed that students not involved in bullying reported lower self-criticism (i.e., higher scores on the “low self-judgment” component) than victims and bully/victims (*ps* < 0.001), and observers reported lower self-criticism (i.e., higher scores on the “low self-judgment” component) than victims (*p* = .033) and bully/victims (*p* = .006). For “Self-Kindness”, observers scored higher than bully/victims (*p* = .021).

Although the multivariate role × gender interaction was statistically significant, univariate interaction effects were not. Exploratory simple effects suggested that role differences were more pronounced among girls than boys. These findings should be interpreted cautiously given unequal cell sizes (notably girls–bullies *n* = 12).

### Associations Between PTSD Symptoms, Mental Resilience, and Self-compassion

As shown in Table 3, overall mental resilience was positively correlated to overall self-compassion and its components “self-kindness”, “common humanity”, and “mindfulness”. The subscale of mental resilience, “personal strength and structured style”, was significantly positively associated to overall self-compassion and its four components. Moreover, the subscale of mental resilience, “self-efficacy and ability to achieve”, was also significantly positively associated to the overall self-compassion and its components “self-kindness”, “common humanity”, and “mindfulness”.

**Table 3:** Correlations Between Post-traumatic Stress, Mental Resilience, and Self-Compassion

1. Post-traumatic stress	1	2	3	4	5	6	7	8	9	10	11
	1.00										
2. Intrusive thoughts, numbing, hyperarousal	0.94^**^	1.00									
3. Negative rumination	0.84^**^	0.71^**^	1.00								
4. Mental resilience	− 0.13**	− 0.25^**^	− 0.05	1.00							
5. Personal strength and structured style	− 0.19**	− 0.29^**^	− 0.13^**^	0.96^**^	1.00						
6. Self-efficacy, ability to achieve	− 0.01	− 0.13^**^	0.11^**^	0.89^**^	0.73^**^	1.00					
7. Self-compassion	− 0.32^**^	− 0.35^**^	− 0.31^**^	0.42^**^	0.50^**^	0.22^**^	1.00				
8. Low self-judgment	− 0.42^**^	− 0.38^**^	− 0.46^**^	0.01	0.10^*^	− 0.15^**^	0.64^**^	1.00			
9. Self-kindness	0.02	− 0.05	0.08^*^	0.51^**^	0.51^**^	0.43^**^	0.56^**^	− 0.21^**^	1.00		
10. Common humanity	0.11^**^	0.05	0.17^**^	0.37^**^	0.35^**^	0.33^**^	0.37^**^	− 0.37^**^	0.66^**^	1.00	
11. Mindfulness	0.04	− 0.05	0.09^*^	0.48^**^	0.47^**^	0.42^**^	0.47^**^	− 0.23^**^	0.68^**^	0.65^**^	1.00

Note: *p < .05 (2-tailed), **p < .01 (2-tailed).1: Post-traumatic stress, 2: Intrusive thoughts, numbing, hyperarousal, 3: Negative rumination, 4: Mental resilience, 5: Personal strength and structured style, 6: Self-efficacy, ability to achieve, 7: Self-compassion, 8: Low Self-Judgment, 9: Self-kindness, 10: Common humanity, 11: Mindfulness.

Additionally, the subscale of PTSD, “negative rumination”, was also significantly positively associated to mental resilience subscale, “self-efficacy and ability to achieve”. PTSD subscale, “negative rumination” was also significantly positively associated to self-compassion components, “self-kindness”, “common humanity” and “mindfulness”. Finally, overall PTSD was also significantly positively associated to the self-compassion component, “common humanity”.

Overall PTSD was negatively correlated to overall mental resilience scale and its subscale “personal strength and structured style”. Moreover, overall PTSD was negatively correlated to overall self-compassion and its component, “low self-judgment”. “Low self-judgment” was reverse-scored; therefore, negative correlations with “low self-judgment” indicate higher self-criticism (i.e., greater self-judgment).

The subscale of PTSD, “intrusive thoughts, numbing and hyperarousal”, was negatively correlated to overall mental resilience and its two subscales. The subscale of PTSD, “negative rumination”, was negatively correlated to mental resilience subscale, “personal strength and structured style”.

The subscales of PTSD, “intrusive thoughts, numbing and hyperarousal” and “negative rumination”, were negatively correlated to overall self-compassion and its component, “low self-judgment”. Finally, the subscale of mental resilience, “self-efficacy and ability to achieve”, was negatively correlated to self-compassion component, “low self-judgment”.

## Discussion

This study extends prior research by examining gender, and bullying-role differences in adolescents’ PTSD symptoms, mental resilience, and self-compassion, and by exploring associations among these constructs. These findings align with conceptualizations of bullying as an unwanted interpersonal behavior that can have trauma-like sequelae for youth (Evans et al., 2017; Moore et al., 2017), and with the view that bullying-related distress may be embedded in a broader social process shaped by peer norms (O’Higgins Norman et al., 2024).

Overall, the study hypotheses were partially supported. Consistent with Hypothesis 1, higher total PTSD symptoms were associated with lower mental resilience and lower self-compassion, and mental resilience was positively associated with self-compassion. In line with Hypothesis 2, girls reported higher PTSD symptom levels than boys. Hypothesis 3 received mixed support: boys reported higher total self-compassion, with the clearest gender difference observed for low self-judgment, whereas no gender differences were observed for overall resilience and girls scored higher on the resilience component “self-efficacy and ability to achieve”. Supporting Hypothesis 4, victims and bully/victims reported higher PTSD symptoms than bullies, observers, and non-involved students. Finally, Hypothesis 5 was partially supported: observers and non-involved students generally showed higher self-compassion than victims and bully/victims, whereas resilience differences were most pronounced for observers (rather than non-involved students), particularly on “personal strength and structured style” and “self-efficacy/ability to achieve”.

Concerning gender differences, in line with evidence from previous studies (Idsoe et al., 2012), girls exhibited greater PTSD symptoms, “intrusive thoughts, numbing, hyperarousal”, and “negative rumination”, than boys. This pattern may reflect gender-linked socialization processes that influence the appraisal and reporting of distress. In addition, the gender × role interaction for “intrusive thoughts, numbing, and hyperarousal” indicated that girls reported higher scores than boys specifically among victims and bully/victims, suggesting that gender differences may be more pronounced in groups with direct exposure to bullying-related adversity.

Concerning self-compassion, boys reported higher levels than girls.

This gender difference was most evident for low self-judgment (i.e., lower self-criticism), with boys reporting higher scores than girls. This result is consistent with those of Yan et al. (2022) concerning that only males were found to reduce depression levels through self-compassion. Yan et al. (2022) supported that when females experience bullying, they may blame themselves and therefore cannot further suppress depression symptoms through self-compassion. Notably, the gender difference in self-compassion was qualified by a significant gender × role interaction: boys reported higher self-compassion than girls among victims and, more prominently, among bully/victims.

Additionally, in contrast with previous literature (Andreou et al., 2020; Saaduddin et al., 2023), girls scored higher than boys on the resilience component ‘self-efficacy and ability to achieve”, whereas no gender differences emerged for overall resilience or for “personal strength and structured style”. One possible interpretation is that boys and girls may draw on different coping resources when faced with bullying-related stress. However, given the cross-sectional design, these patterns should be interpreted cautiously. Prior work suggests that self-efficacy and resilience can partially mediate the association between bullying experiences and mental health outcomes (Lin et al., 2020).

Concerning PTSD symptoms (intrusive thoughts, numbing, hyperarousal and negative rumination), our findings are consistent with prior research (Idsoe et al., 2012; Kennedy, 2021). Victims and bully/victims reported higher levels of PTSD symptoms than bullies, observers, and non-involved students. In other words, bully/victims resembled victims in PTSD symptomatology (Andreou et al., 2021; Sterzing et al., 2020), and both groups appeared to be the most burdened in terms of PTSD symptoms. More specifically, within the victim and bully/victim groups, girls reported higher levels of intrusive thoughts, numbing, and hyperarousal than boys. This pattern is consistent with the broader profile of observers, who scored higher than victims and bully/victims on the “personal strength and structured style*”* component of resilience, as well as with the comparatively higher self-compassion reported by observers and non-involved students— lower self-criticism (i.e., higher scores on the “low self-judgment” component) relative to victims and bully/victims. In addition, observers reported higher “self-kindness” than bully/victims.

In the resilience domain, bullies scored significantly higher than bully/victims on *“*personal strength and structured style*”*, whereas observers scored significantly higher than bully/victims and non-involved students on “self-efficacy/ability to achieve*”*. Overall, these findings suggest that more complex involvement in bullying, particularly bully/victim status, may be associated with comparatively lower resilience-related personal resources.

Consistent with this pattern, bully/victims reported significantly lower self-compassion than bullies, indicating that less complex roles may be associated with comparatively higher self-compassion. Similarly, Bunnett (2021) reported lower resilience among bully/victims compared to bullies. Moreover, Kennedy (2021) highlights meaningful heterogeneity among bully/victims, suggesting that greater dual involvement may be linked to higher trauma symptoms. However, given the cross-sectional design, these patterns should be interpreted as associations rather than evidence of protective or causal effects.

Moreover, the results confirmed the positive association between mental resilience and self-compassion, found in previous studies (Bowornkittikun et al., 2021). This suggests that adolescents with a high level of self-compassion are more likely to have a high level of resilience. Therefore, adolescents with more “personal strength, structured coping style”, “belief in their self-efficacy and ability to achieve”, would demonstrate inner support regarding their experiences of bullying, successfully cope with painful thoughts and feelings, and recognize that these thoughts and feelings connect rather than separate them from other peers.

At the same time, the negative association between the resilience component “self-efficacy and ability to achieve” and the self-compassion component “low self-judgment” suggests that, for some adolescents, a stronger sense of capability or achievement orientation may coexist with a more self-demanding stance. Given the direction of scoring and the small correlational pattern observed, this finding should be interpreted cautiously.

Additionally, the results showed that PTSD was negatively associated with mental resilience and self-compassion. Given the cross-sectional design, these findings should be interpreted as co-occurring differences rather than evidence of protective or causal effects. This pattern is consistent with prior evidence linking bullying involvement to trauma symptoms and to lower self-compassion/resilience (Chu et al., 2018; Fang et al., 2022; Xu et al., 2024). Longitudinal research is needed to clarify directionality (e.g., whether bullying-related distress undermines self-compassion over time and/or whether self-compassion and resilience buffer subsequent trauma symptoms).

Finally, PTSD was negatively correlated to “low self-judgment”. This pattern is consistent with conceptualizations of self-compassion as an adaptive coping resource (Neff, 2003) and with evidence linking bullying victimization to heightened shame and self-criticism and to processes that may undermine self-compassion (Beduna & Perrone-McGovern, 2019; Múzquiz et al., 2023; Xu et al., 2024; Zăbavă, 2020). Given that “low self-judgment” was reverse-scored, this negative association indicates that higher PTSD symptoms co-occurred with greater self-criticism. This is compatible with shame/self-blame processes observed following victimization experiences and may represent a promising intervention target alongside resilience-building strategies.

Although the overall pattern linking PTSD with resilience and self-compassion was predominantly negative, a few small positive correlations emerged for the PTSD component “negative rumination.” Specifically, “negative rumination” was positively associated with the resilience component “self-efficacy and ability to achieve” and with the self-compassion components “self-kindness,” “common humanity,” and “mindfulness.” A similarly small positive association was also observed between overall PTSD and the self-compassion component “common humanity.” Given their small magnitude and the cross-sectional design, these associations should be interpreted cautiously. This pattern is broadly consistent with prior empirical work suggesting that PTSD symptoms may coexist with more adaptive processes following school bullying experiences, including resilience and post-traumatic growth (Andreou et al., 2021; Makrydaki et al., 2024). One possible interpretation is that some adolescents who experience repetitive negative thoughts may nevertheless mobilize certain coping-related and self-compassionate resources, such as perceived self-efficacy, balanced awareness, and a sense of shared human experience.

### Limitations and Future Directions

Several limitations should be considered when interpreting these findings. First, the cross-sectional design does not permit conclusions about directionality or causality among bullying roles, PTSD symptoms, resilience, and self-compassion. Longitudinal designs are needed to clarify temporal ordering and to examine whether changes in resilience and self-compassion prospectively predict changes in trauma-related symptoms following bullying exposure. Second, all variables were assessed via adolescent self-report, which may be affected by shared method variance, social desirability, and recall biases. Future studies should incorporate multi-informant (e.g., peer, teacher, parent) and/or multi-method approaches (e.g., clinical interviews, school records) to strengthen measurement validity.

Furthermore, one PTSD subscale (“changes in daily life”) showed relatively low internal consistency (α = 0.52). Although this subscale was excluded from subsequent subscale-level analyses and was not interpreted further, this psychometric limitation should be considered when interpreting PTSD subscale-level findings.

In addition, to clarify multivariate findings we conducted separate MANOVAs for subscales and multiple follow-up tests. Although Bonferroni adjustments were applied, the number of comparisons may still increase the risk of Type I/Type II error and some effects may be over- or under-estimated. Replication with preregistered analytic plans and/or more integrated multivariate models is warranted.

Third, participants were recruited from the first grade of high schools in one region of Greece (Thessaly). Although the sample size was adequate, the findings may not generalize to adolescents from other regions, age groups, or cultural contexts. Replication across diverse samples is warranted.

Fourth, the present study operationalized bullying involvement using role-based categories. Future research could examine the frequency, duration, and types of bullying (e.g., physical, verbal, relational, cyber) as well as contextual factors (e.g., classroom climate, teacher response, peer norms) that may shape trauma-related outcomes.

Finally, future work should extend beyond mean group differences to test theoretically informed mechanisms (e.g., mediation) and boundary conditions (e.g., moderation) involving resilience and self-compassion. For example, resilience and self-compassion may buffer the association between victimization and PTSD symptoms, and these effects may vary by gender or by bullying role.

### Practical and Clinical Implications

The present findings highlight potential targets for prevention and support efforts in school settings. Given the elevated PTSD symptoms observed among victims and bully/victims, early identification and access to school-based psychological support may be particularly important for these groups. At the same time, the higher resilience and self-compassion observed among observers and non-involved students suggest that fostering adaptive coping resources may be beneficial at the classroom and whole-school level. Interventions that include components aimed at strengthening resilience (e.g., structured coping and self-efficacy) and cultivating self-compassion (e.g., self-kindness and mindful awareness) may complement established anti-bullying programs. Finally, bystander-focused approaches that empower observers to respond safely and effectively may help improve the broader school climate.

## Conclusion

In conclusion, resilience and self-compassion were associated with lower PTSD symptom levels among adolescents involved in school bullying roles. Given the cross-sectional design, these findings should not be interpreted as evidence of causality; however, they highlight potentially modifiable resources that may be useful targets in school-based prevention and support efforts. Interventions may benefit from considering bullying role and gender differences when tailoring support, with an emphasis on strengthening adaptive coping resources (e.g., self-efficacy/structured coping) and self-compassionate responding (e.g., reducing self-criticism and fostering self-kindness).

## Data Availability

The data presented in this study are available upon request to the corresponding author.

## Abbreviations

PTSD: Post-traumatic stress disorder
